# Identification of prognostic inflammatory factors in colorectal liver metastases

**DOI:** 10.1186/1471-2407-14-542

**Published:** 2014-07-28

**Authors:** Trevor D Hamilton, Derek Leugner, Karen Kopciuk, Elijah Dixon, Francis R Sutherland, Oliver F Bathe

**Affiliations:** Department of Surgery, University of Calgary, Calgary, AB Canada; Department of Mathematics and Statistics, University of Calgary, Calgary, AB Canada; Department of Oncology, University of Calgary, 1331-29th St NW, Calgary, T2N 4N2 AB Canada

**Keywords:** Colorectal, Cancer, Liver, Metastases, Inflammatory, CRP, Cytokine, Prognostic, Surgery

## Abstract

**Background:**

The modified Glasgow Prognostic Score (mGPS) has been reported to be an important prognostic indicator in a number of tumor types, including colorectal cancer (CRC). The features of the inflammatory state thought to accompany elevated C-reactive protein (CRP), a key feature of mGPS, were characterized in patients with colorectal liver metastases. Additional inflammatory mediators that contribute to prognosis were explored.

**Methods:**

In sera from 69 patients with colorectal liver metastases, a panel of 42 inflammatory mediators were quantified as a function of CRP levels, and as a function of disease-free survival. Multivariate statistical methods were used to determine association of each mediator with elevated CRP and truncated disease-free survival.

**Results:**

Elevated CRP was confirmed to be a strong predictor of survival (HR 4.00, p = 0.001) and recurrence (HR 3.30, p = 0.002). The inflammatory state associated with elevated CRP was comprised of raised IL-1β, IL-6, IL-12 and IL-15. In addition, elevated IL-8 and PDGF-AB/BB and decreased eotaxin and IP-10 were associated with worse disease-free and overall survival.

**Conclusions:**

Elevated CRP is associated with a proinflammatory state. The inflammatory state is an important prognostic indicator in CRC liver metastases. The individual contributions of tumor biology and the host to this inflammatory response will require further investigation.

**Electronic supplementary material:**

The online version of this article (doi:10.1186/1471-2407-14-542) contains supplementary material, which is available to authorized users.

## Background

Colorectal cancer (CRC) is the second leading cause of cancer death in the western world. The liver is the most common site of metastases, affecting approximately 25–35% [1]. Liver metastases are the most common cause of death in CRC patients [1]. Although there are a number of therapies for treating metastatic CRC isolated to the liver, including ablative therapies and systemic chemotherapy, resection remains the mainstay of treatment for potential cure. With resection, survivals have steadily improved with advances in surgical technique and systemic therapy. In more recent series, median survival is now greater than 50 months and 5-year survival exceeds 50% [2–4].

With the expansion of therapeutic options, it is becoming increasingly important to select the best treatment strategy comprising various sequences of systemic therapy and surgery. For example, if a subset of individuals is known to have short disease-free survivals after resection, systemic therapy may play a more important role, either instead of resection or as an adjunct to resection. To this end, clinical risk scores have been developed to stratify patients by prognosis [5–13]. Clinical risk scores are easily employed because the data are readily available from standard preoperative tests employed in clinical practice. However, they shed little insight on the underlying biology of the disease, which is integral to the formulation of new therapeutic strategies.

The modified Glasgow Prognostic Score (mGPS) [14] is a rudimentary means to derive a picture of the overall biological state. The mGPS, based on serum C-reactive protein (CRP) and albumin, was described by McMillan and coworkers [15]. Several series have demonstrated that mGPS effectively stratifies patients by prognosis in CRC [16, 17], including CRC liver metastases [18–21]. mGPS also effectively prognosticates in cancers of the lung, esophagus, pancreas, breast, ovary, bladder and kidney [22–28]. mGPS is thought to reflect the inflammatory state, although the nature of the inflammatory state has not yet been characterized, and the contributions of tumor and host to this inflammatory state have not been delineated.

Understanding the underlying biology of mGPS may leverage its use as a tool to make treatment decisions. That is, with a better comprehension of the inflammatory pathways associated with poor clinical outcomes, it may be possible to redirect the inflammatory response to a more favorable course. The aim of the present study was to characterize the inflammatory state associated with elevated CRP, the main parameter influencing mGPS. A panel of 42 inflammatory mediators, including cytokines and chemokines, were evaluated as a function of CRP. In addition, other important prognostic inflammatory mediators were identified, which may further refine any inflammation-based prognostic scoring system.

## Methods

This study was approved by the Conjoint Health Research Ethics Board at the University of Calgary (Ethics ID E21805). Clinically annotated serum samples were collected prospectively from consented patients who underwent surgery for resection of liver metastases. All patients were treated at the Foothills Medical Centre, a tertiary referral centre, between 2006 and 2011. Patients with extrahepatic disease, or any acute inflammation or sepsis were specifically excluded. Surgical pathology was reviewed for all patients, and confirmed all had colorectal adenocarcinoma. Samples were collected in a plastic gold top Vacutainer tube (BD Biosciences), which contained a clot activator and a gel for serum separation. Samples were processed within 6 hours of collection, then frozen at -20°C until the time of analysis. All samples were collected from patients who had fasted, prior to surgery.

The inflammatory state was characterized in sera using a multiplexed protein bead assay (Luminex, Austin, TX), which measured 42 different inflammatory mediators. Two cytokines (IL-3 and IL-9) were removed from the subsequent analysis due to their high number of zero values (69/70 and 68/70, respectively).

### Data analysis

Patients were allocated to one of two groups, based on a CRP cutpoint level of 10 mg/L, as reported by others [14–16, 18, 19]. Descriptive statistics were utilized to characterize the clinical parameters of groups: unpaired *t*-tests with unequal variances assumed (Welch’s *t*-test) were used to compare means, and Fisher’s exact tests were used to compare categorical variables. Quarter-root transformations were employed to improve data symmetry while preserving zero scores assigned to cytokine values below the assay detection limit. All tests of significance were 2-sided and a *p*-value less than 0.05 was considered *a priori* to represent statistical significance between groups of patients in these univariate analyses.

To identify individual cytokines associated with elevated CRP, two-sample *t*-tests as well as penalized logistic regression methods were applied based on the elastic net approach [29, 30]. Multiple testing adjustment for the two-sample *t*-tests was taken into account with the False Discovery Rate via the Significance Analysis Microarrays (SAM) program, which also provides an estimated *p*-value cutoff for a specified false discovery rate [31].

Kaplan-Meier survival plots and Cox Proportional Hazards regression models were used to evaluate the association between cytokines and disease-specific survival. The primary endpoints for that analysis were disease-free survival and overall survival, calculated from date of surgery. Patients without recurrence were censored at their date of last follow-up.

## Results

### Inflammatory mediators associated with elevated CRP

Serum inflammatory mediators were evaluated in 70 patients who were considered for surgery. One patient who underwent resection had a recent tooth infection at time of serum sample collection and consequently was excluded from analysis. CRP was measured in the 69 patients with CRC liver metastases and the inflammatory profile was evaluated to determine the nature of the inflammatory response associated with elevated CRP.

The median age for the entire cohort was 61 years (range 29–86), and of those assessed there were 47 males (68%) and 22 females (32%). The median CEA was 5.0 (range 0.6–682). Liver metastases were synchronous in 36 patients (52%). Of those patients with synchronous liver metastases, all had undergone resection of the primary tumor prior to liver resection (and prior to blood collection); no patients were treated with simultaneous colon and liver resections.

A total of 40 inflammatory mediators were tested for their association with elevated CRP (Additional file 1: Table S1). By univariate analysis, there were 4 cytokines that were strongly associated with an elevated CRP: IL-1β, IL-6, IL-12, and IL-15 (Table 1). After adjusting for multiple hypothesis testing using the false discovery rate (FDR), 3 cytokines, IL-1β, IL-12, and IL-15 were found to be significantly associated with elevated CRP (Table 1).

**Table 1:** **Cytokines significantly associated with elevated CRP using univariate and multiple hypothesis testing analysis**

Cytokine	Univariate analysis (p)	SAM (q)
IL-1β	0.023	0.000
IL-6	0.004	0.636
IL-12	0.041	0.000
IL-15	0.006	0.000

IL - Interleukin.

SAM - Significance analysis of microarrays.

### Prognostic inflammatory profile in resected liver metastases

To understand which inflammatory mediators were of biological significance, we evaluated mediators associated with recurrence and overall survival. For this analysis, only patients that had a resection were evaluated. Of the 69 patients initially assessed, four patients had progression of disease and did not proceed to surgery, and 13 patients were found to have unresectable disease; these were excluded from further analysis. The baseline clinical and pathologic characteristics of patients in the study who had a liver resection are represented in Table 2. A total of 27 patients (52%) had a normal CRP (<10 mg/L) and 25 patients had an elevated CRP (≥10 mg/L). Liver resection was performed in 34 males (65%) and 18 females (35%). The median age was 61 years (range 29–86). Neoadjuvant chemotherapy was administered in 28 patients (54%), adjuvant chemotherapy was administered in 30 patients (58%). Median CEA was 3.2 (range 0.6–134). Liver metastases were synchronous in 28 patients (54%). In 43 patients (83%), R0 resections were achieved; the remainder had positive microscopic margins. Solitary metastases were resected in 50%; up to 7 lesions were resected in any individual. The median tumor size was 3.5 cm (range 0.5–9.0). There was no significant difference in any of these factors between the group with a normal CRP and the group with elevated CRP (Table 2). Specifically, CRP levels did not vary as a function of chemotherapy, CEA level, or number and size of metastases.

**Table 2:** **Clinical and pathologic characteristics of resected patients with normal and elevated CRP levels**

	CRP < 10		CRP ≥ 10		
	n	%	n	%	P-value
Age (years)					
< 65	15	55.6	15	60.0	0.785
≥ 65	12	44.4	10	40.0	
Gender					
Male	18	66.7	16	64.0	1.000
Female	9	33.3	9	36.0	
Neoadjuvant chemo					
Yes	13	48.1	15	62.5	0.400
No	14	51.9	9	37.5	
Adjuvant chemo					
Yes	16	66.7	14	66.7	1.000
No	8	33.3	7	33.3	
CEA (ng/mL)					
< 20	15	68.2	19	79.2	0.508
≥ 20	7	31.8	5	20.8	
Disease-free interval (months)					
< 12	21	77.8	14	56.0	0.140
≥ 12	6	22.2	11	44.0	
Resection margin					
Negative	25	92.6	18	72.0	0.071
Positive	2	7.4	7	28.0	
Number of tumors					
1	14	53.8	12	54.5	1.000
> 1	12	46.2	10	45.5	
Largest tumor size (cm)					
< 5	22	84.6	15	68.2	0.301
≥ 5	4	15.4	7	31.8	

CRP - C-Reactive Protein.

CEA - Carcinoembryonic Antigen.

Some missing data.

**Figure 1:**
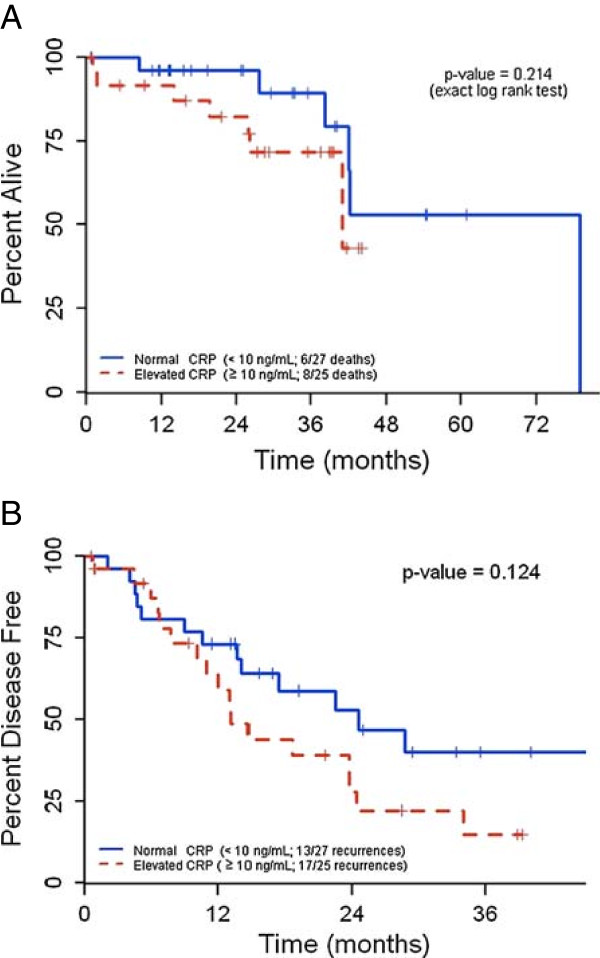
**Kaplan-Meier curves depicting survival as a function of CRP levels. A**. Overall survival as a function of serum CRP levels. **B**. Disease-free survival as a function of serum CRP levels. Groups with elevated and normal CRP levels were compared by log rank test.

Median follow-up for patients still alive was 20 months (range 1–49 months). During follow-up, 15 patients (28%) died, and 31 patients (58%) developed recurrent disease. The median overall survival in patients with normal and elevated CRP was 79 months and 41 months, respectively (Figure 1A). However, this difference was not statistically significant by log rank test. The median disease-free survival in patients with normal CRP was 23 months and, in patients with elevated CRP, was 13 months (Figure 1B). Again, this was not statistically significant by log rank test, due to the small sample size. However, when CRP was analyzed as a continuous variable (as opposed to a binary variable), it was found to be a strong predictor of overall survival (hazard ratio 4.00; Table 3) and disease-free survival (hazard ratio 3.30, Table 4).

**Table 3:** **Inflammatory mediators significantly predictive of median overall survival**

Cytokine	Hazard ratio	95% CI	P-value
CRP	4.00	1.70–9.31	0.001
IL-8	4.96	1.35–17.6	0.014
PDGF-AB/BB	3.16	1.42–6.90	0.005

CRP - C-Reactive Protein.

PDGF - Platelet Derived Growth Factor.

**Table 4:** **Inflammatory mediators significantly predictive of median disease-free survival**

Cytokines	Hazard ratio	95% CI	P-value
CRP	3.30	1.56–7.00	0.002
Eotaxin	0.28	0.11–0.79	0.014
IP-10	0.41	0.17–0.96	0.041

CRP - C-Reactive Protein.

IP - Interferon inducible-protein.

Finally, we sought to determine if any other inflammatory changes (in addition to those associated with elevated CRP) were prognostic. Elevated levels of IL-8 and PDGF-AB/BB were associated with a worse overall survival by univariate analysis (Table 3). After adjusting for multiple hypothesis testing, only CRP and PDGR-AB/BB were significant. In contrast, elevated levels of eotaxin and IP-10 were associated with lower rates of recurrence (Table 4). However, after adjusting for multiple hypothesis testing, only CRP was significantly associated with recurrence.

## Discussion

As therapeutic options become more numerous, it becomes increasingly important to develop tools to select the appropriate treatment regimen. In the case of resectable liver metastases, clinical risk scores (employing factors such as CEA, size and number of metastases, disease-free interval, nodal status, margins) effectively stratify most patients by risk of recurrence and survival [5–13]. When encountering a patient with a high-risk score, it may prompt the clinician to defer surgery and/or administer systemic therapy. Similarly, the mGPS has been described to effectively prognosticate for CRC and for CR liver metastases [16–21]. Indeed, our small series confirms that CRP levels relate to disease-free and overall survival. mGPS is relatively simple to apply and presumably reflects underlying tumor and/or host biology, although it is not entirely clear how this will affect clinical management.

mGPS is thought to reflect the inflammatory response secondary to malignancy, although the inflammatory response has not been confirmed or characterized. Others have described changes in circulating inflammatory mediators associated with CRC, including elevated IL-2, IL-6, and IL-8 without evaluating whether these relate to mGPS [32–34]. More recently, McMillan’s group has linked mGPS with IL-6 and tumor necrosis in patients with primary resected colorectal cancer [35]. The current study extends on that work in that a more comprehensive panel of inflammatory mediators was assessed as a function of mGPS. We have demonstrated that elevated CRP is associated with increases in IL-1β, IL-6, IL-12 and IL-15 by univariate analysis. All of these cytokines with the exception of IL-6 remained significantly associated with high CRP levels on multivariate analysis. In general, this confirms that a high mGPS is reflective of a proinflammatory state, with features of a Th1 response.

In addition, a number of other inflammatory mediators (elevated IL-8 and PDGF-AB/BB, as well as depressed eotaxin and IP-10) were found to be independently associated with increased recurrence or truncated survival. IL-8 is a proinflammatory chemokine but is also overexpressed on metastatic colon cancer cells [36], as well as other cancer cells [37]. In the context of cancer, IL-8 is proangiogenic, attracts neutrophils to tumor, and has a number of other functions that encourage tumor growth and metastasis. It may also confer chemoresistance [37]. Colon cancer is associated with elevated platelet concentrations of PDGF [38], presumably reflecting a proangiogenic state. Its expression has not been previously reported to discriminate biologically meaningful subsets. Eotaxin, a chemokine that recruits eosinophils, has been reported to be expressed at higher levels in CRC compared to normal tissue [39, 40], although circulating eotaxin is decreased in CRC patients compared to disease-free controls and progressively decreases with more advanced disease [40]. IP-10 is a chemokine that plays a role in the chemoattraction of monocytes, endothelial cells and fibroblasts; it is also an inhibitor of angiogenesis. It is inversely correlated with growth and metastasis in experimental models [41, 42]. One previous study has shown that low circulating levels of IP-10 correlate with poor prognosis in CRC [43].

In our analysis of cytokines related to prognosis, we did not formally take into account the effect of clinicopathological factors because we could not detect any relationship between CRP levels and because of the limited sample size. One potential confounding factor in our series is the administration of chemotherapy; approximately half of patients had neoadjuvant chemotherapy. Interestingly, there was no difference in CRP levels in patients who did and did not receive chemotherapy. In fact, none of the clinical or pathological factors (known to influence prognosis) that we examined had a significant association with elevated CRP. Therefore, it is unlikely that such confounding factors influenced our survival analysis. However, in future studies, it will be important to validate the refined prognostic cytokine profile in a larger group of patients undergoing resection of colorectal liver metastases. In addition, the independent contribution of this panel of biomarkers on outcomes will have to be evaluated with a multivariable survival analysis.

In addition to mGPS, a number of other indices of inflammation have been described, including neutrophil/lymphocyte ratio (NLR), platelet/lymphocyte ratio (PLR), and prognostic nutritional index (PNI; a combination of albumin and lymphocyte count). It is presently unclear whether all of these indices represent the same type of inflammatory response (characterized by the same pattern of circulating cytokines). Moreover, it is not known what indices best reflect the inflammatory state associated with a poor prognosis. This may well vary between types of malignancies. In a group of pancreatic cancers with various stages of disease and undergoing diverse treatments, NLR was prognostic and other indices were not [44]. In hepatocellular carcinoma, mGPS performed better as a prognosticating tool than NLR and PNI [45]. This discrepancy may be related to differences in the nature of the inflammatory response associated with different malignancies. Therefore, further study is required to understand the molecular and disease-related causes of cancer-associated inflammation, including how that inflammatory state may differ between cancers.

An important question related to any studies focusing on prognostic factors for cancer is whether any factor that effectively categorizes individuals could be used for treatment stratification. For example, for primary colon cancer, adjuvant chemotherapy is recommended for stage III, and only high risk patients in stage II are typically considered for adjuvant therapy [46]. Similarly, many clinicians would advocate administration of neoadjuvant chemotherapy for high risk patients with colorectal liver metastases as a means to better select surgical candidates [47]. While higher risk patients have a greater likelihood of benefiting from systemic therapy, in our view, treatment stratification based on prognostic biomarkers is not the ultimate goal. Rather, efforts must be directed at understanding the underlying biology that leads to poor oncologic outcomes so that novel therapeutic targets can be identified.

While the cohort studied was small, our data confirm that mGPS is a reflection of the inflammatory state. Specifically, elevated CRP indicates a proinflammatory state with features of a Th1 response. We have also demonstrated that prognosis of resected CR liver metastases is affected by a number of additional inflammatory (and perhaps angiogenic) events. This study paves the way to more detailed (and targeted) interrogations on the role of inflammation in the biology of CRC. In particular, it will be important to understand the contributions of the molecular features of tumor that incite a proinflammatory response, as well as characteristics of the host which perhaps lead to susceptibility to inflammation. The host response associated with elevated CRP (including the metabolic response and effects on distant organs) must be better understood, to gain an understanding of why the proinflammatory state is so detrimental. These more advanced studies will not only add to our capability to prognosticate; understanding the molecular pathways that portend a poor prognosis may lead to development of novel therapeutic strategies.

In all, our studies adhere to the REMARK reporting guidelines for prognostic tumor markers [48].

## Conclusions

In patients with colorectal liver metastases, elevated CRP is associated with increased circulating proinflammatory cytokines with features of a Th1 response. Elevated CRP is associated with a shorter disease-free and overall survival following resection. Other cytokines that influenced survival outcomes included IL-8, PDGF-AB/BB, eotaxin and IP-10.

## Electronic supplementary material

Additional file 1: Table S1: Mean and standard deviation of all inflammatory mediators analyzed. (PDF 44 KB)
