# Supplementary material for: Identification of prognostic inflammatory factors in colorectal liver metastases
Source: BMC Cancer. 2014 Jul 28;14:542. doi: 10.1186/1471-2407-14-542 (PMC4125702; doi:10.1186/1471-2407-14-542)
Supplement: Supplementary file 1 — Additional file 1: Table S1: Mean and standard deviation of all inflammatory mediators analyzed. (PDF 44 KB) [file 12885_2013_4734_MOESM1_ESM.pdf]

Supplementary Table 1 Mean and standard deviation of all inflammatory mediators analyzed

	CRP < 10		CRP ≥ 10	
	Mean	SD	Mean	SD
Epidermal Growth Factor (EGF)	40.61	39.06	40.89	36.04
Eotaxin	114.49	91.37	88.25	43.31
Fibroblast Growth Factor 2 (FGF-2)	24.35	23.22	31.72	27.27
FMS Related Tyrosin Kinase 3 Ligand (Flt-3 Ligand)	18.33	12.70	22.78	21.20
Fractalkine	68.89	140.69	49.91	62.52
Granulocyte Colony-Stimulating Factor (G-CSF)	7.25	12.05	6.22	11.25
Granulocyte Macrophage Colony-Stimulating Factor (GM-CSF)	19.43	39.13	32.62	58.87
Growth Related Oncogene (GRO)	457.67	180.97	540.84	288.51
Interferon alpha 2 (IFNa2)	13.74	34.77	9.27	14.73
Interferon gamma (IFNg)	20.32	47.95	20.64	20.32
Interleukin 1 alpha (IL-1a)	7.90	24.63	2.35	7.02
Interleukin 1 beta (IL-1b)	12.90	41.01	15.46	39.16
Interleukin 1 Receptor Antagonist (IL-1ra)	17.84	49.04	14.23	29.57
Interleukin 2 (IL-2)	9.41	25.34	19.37	40.10
Interleukin 4 (IL-4)	14.59	23.07	20.35	42.52
Interleukin 5 (IL-5)	0.85	3.71	0.41	0.83
Interleukin 6 (IL-6)	4.81	9.89	6.91	5.82
Interleukin 7 (IL-7)	4.31	3.01	6.28	5.88
Interleukin 8 (IL-8)	25.19	24.51	34.37	50.85
Interleukin 10 (IL-10)	5.93	11.59	4.98	4.91
Interleukin 12 (IL-12)	45.67	113.34	49.87	118.14
Interleukin 13 (IL-13)	3.49	14.94	2.01	3.75
Interleukin 15 (IL-15)	17.42	52.59	16.55	25.31
Interleukin 17 (IL-17)	5.08	11.78	5.61	7.63
Interferon Gamma-Induced Protein 10 (IP-10)	94.87	60.22	111.28	77.61
Monocyte Chemotactic Protein 1 (MCP-1)	495.29	242.77	482.79	167.40
Monocyte Chemotactic Protein 3 (MCP-3)	29.66	59.73	29.81	37.31
Macrophage Derived Chemokine (MDC)	1400.66	689.60	1489.90	699.31
Macrophage Inflammatory Protein 1 alpha (MIP-1a)	56.89	93.70	58.80	74.75
Macrophage Inflammatory Protein 1 beta (MIP-1b)	78.74	165.86	67.70	79.01
Platelet-Derived Growth Factor AA (PDGF-AA)	3028.06	4316.98	3851.61	5208.67
Platelet-Derived Growth Factor AB/BB (PDGF-AB/BB)	8348.14	3439.21	9166.27	3412.84
Regulated and normal T cell expressed and secreted (RANTES)	1291.78	614.28	1324.76	679.78
CD 40 Ligand (CD40L)	4963.40	2707.31	4877.01	2537.04
Soluble Interleukin 2 Receptor alpha (sIL-2Ra)	26.51	47.75	20.06	26.10
Transforming Growth Factor alpha (TGFa)	12.95	15.43	11.27	8.54
Tumor Necrosis Factor alpha (TNFa)	4.15	7.19	3.73	2.49
Tumor Necrosis Factor beta (TNFb)	11.81	48.62	3.70	6.69
Vascular Endothelial Growth Factor (VEGF)	99.27	134.51	68.13	62.37

*all units in pg/mL
